# 超高效液相色谱-四极杆-飞行时间高分辨质谱快速筛查确证化妆品中73种常见禁用物质

**DOI:** 10.3724/SP.J.1123.2021.09010

**Published:** 2022-05-08

**Authors:** Yangjie LI, Jiaying HUANG, Jihui FANG, Zhiye HUANG

**Affiliations:** 广东省药品检验所, 国家药品监督管理局化妆品风险评估重点实验室, 广东 广州 510000; Guangdong Institute for Drug Control, National Medical Products Administration Key Laboratory for Safety Risk Assessment of Cosmetics, Guangzhou 510000, China

**Keywords:** 超高效液相色谱, 四极杆-飞行时间高分辨质谱, 禁用物质, 化妆品, ultra performance liquid chromatography (UPLC), quadrupole-time-of-flight high resolution mass spectrometry (Q-TOF HRMS), prohibited compounds, cosmetics

## Abstract

建立了超高效液相色谱-四极杆-飞行时间高分辨质谱(UPLC-Q-TOF HRMS)同时快速筛查确证化妆品中73种常见禁用物质的方法。样品经饱和氯化钠溶液分散均匀后,采用含0.2%甲酸的乙腈溶液超声提取,50 mg PSA净化,以8000 r/min高速冷冻离心除脂,采用Waters Acquity HSS T3色谱柱(100 mm×2.1 mm, 1.8 μm)分离。采用多反应监测高分辨扫描模式(MRM HR),以保留时间、一级母离子精确质量数、同位素丰度比和二级子离子精确质量数实现化妆品中73种禁用物质的快速筛查和确证,基质匹配外标法定量。实验比较了不同提取溶剂、净化吸附剂、色谱条件和质谱扫描模式对73种禁用物质测定的影响,并考察了膏霜剂和水剂的基质效应。结果表明,73种禁用物质线性关系良好,相关系数(*R*^2^)>0.99;检出限为5~150 μg/kg;定量限为15~450 μg/kg;膏霜剂及水剂两种基质在3个加标水平下的回收率为60.3%~130.3%,日内、日间RSD分别为0.8%~10.0%(*n*=6)和1.1%~15.0%(*n*=3)。日常风险监测中检出磺胺甲基异噁唑、甲基泼尼松、林可霉素、对乙酰氨基酚、甲氧苄啶、阿法骨化醇、倍他米松戊酸酯、溴莫尼定、氯霉素、氯苯那敏、氯倍他索丙酸酯、克罗米通、益康唑、酮康唑、泼尼松醋酸酯和泼尼松,检出含量范围为0.5~1136.1 mg/kg。该方法准确、快速、简便,可用于化妆品中73种常见禁用物质的检测。

## Abstract (en)

A high throughput screening method based on ultra performance liquid chromatography-quadrupole-time-of-flight high resolution mass spectrometry (UPLC-Q-TOF HRMS) was developed for the simultaneous and rapid confirmation of 73 prohibited compounds in cosmetics. The sample was dispersed in a saturated sodium chloride solution and ultrasonically extracted using acetonitrile containing 0.2% (v/v) formic acid. The resultant solution was centrifuged and then cleaned using dispersive solid phase extraction using a primary secondary amine (PSA) sorbent. The purified solution was centrifuged, and the supernatant was filtered through a 0.22 μm membrane before determination. The optimal pretreatment method was determined by comparing the recovery rates obtained using different extraction solvents and different amounts of purifying agents. The chromatographic separation conditions and mass spectrometry scanning mode were also optimized. Chromatographic separation was performed on an Acquity UPLC HSS T3 column (100 mm×2.1 mm, 1.8 μm) with gradient elution using 0.1% (v/v) formic acid aqueous solution and methanol as mobile phases. The eluent from the column was further detected using Q-TOF HRMS with the high resolution multiple reaction monitoring (MRM HR) scanning mode. Retention time, precise mass of parent ion, isotope abundance ratio, and precise mass of fragment ions were the parameters considered for rapid untargeted screening and confirmation. The matrix effects of water- and cream-based cosmetics were investigated. The matrix effects could be addressed using the matrix matched standard curve method. The correlation coefficients for the 73 prohibited compounds were all >0.99 in the corresponding linear concentration range. The limits of detection (LODs) were in the range of 5-150 μg/kg, and the limits of quantification (LOQs) were in the range of 15-450 μg/kg. Average recoveries were in the range of 60.3%-130.3% at three spiked levels, and the intra-day and inter-day precisions were 0.8%-10.0% (*n*=6) and 1.1%-15.0% (*n*=3), respectively. A total of 692 cosmetics samples were screened; 16 positive samples were detected, namely, sulfamethoxazole, meprednisone, lincomycin, 4-acetamidophenol, trimethoprim, alfacalcidol, betamethasone 17-valerate, brimonidine, chloramphenicol, chlorpheniramine, clobetasol propionate, crotamiton, econazole, ketoconazole, prednisone 21-acetate, and prednisone, with content in the range of 0.5-1136.1 mg/kg. The optimized method is accurate, fast, and simple, and it is suitable for the routine detection and rapid screening of common prohibited compounds in cosmetics. In addition, a screening and confirmation library was established for the 650 prohibited compounds using SCIEX OS and Library View software, using information-dependent acquisition (IDA)-MS/MS mode for MS data acquisition. The database contains multiple types of information, including formulas, theoretical exact mass, retention time, precise mass of parent ion, isotope abundance ratio, and fragment ion distribution. The library can be used for the simultaneous and rapid confirmation of prohibited compounds in cosmetics.

化妆品作为日常生活用品,其质量安全影响千家万户。随着生活水平和审美要求的提高,人们对化妆品的需求越来越大,而有些不法商家为达到效果好、见效快、成本低等目的,在化妆品中添加各种激素、抗生素等禁用物质^[1⇓⇓⇓-5]^,严重威胁了消费者的身体健康。化妆品监管部门通报的不合格化妆品问题显示,添加禁用物质和超量使用限用物质是当前化妆品质量安全存在的主要问题。

目前,化妆品中禁用物质的检测主要采用《化妆品安全技术规范》^[6]^和国标^[7,8]^等检测标准。但标准中禁限用物质定性定量采用的液相色谱-三重四极杆质谱(LC-MS/MS)只适用于已有标准物质的已知物分析,且检测通量受四极杆扫描速度的限制,无法实现高通量筛查。同时,低分辨的四极杆质量分析器无法区分质荷比相近的干扰物,易造成假阳性结果。超高效液相色谱-四极杆-飞行时间高分辨质谱(UPLC-Q-TOF HRMS)具有高质量精度、高扫描速度、高通量和全质量数据采集等优势,可以大大提高定性筛查的准确度,配合数据库谱库检索功能可广泛用于样品中未知物筛查和多组分的高通量检测^[9]^。

此外,目前已有的标准和检测方法多是针对化妆品中的糖皮质激素类^[1,4]^、抗生素类^[5]^、抗过敏类^[10,11]^、抗组胺类^[12]^、性激素类^[13]^等单一类别化合物进行检测。但化妆品中实际添加的禁用物质种类繁多,检测过程中需要多个标准或检测方法组合使用,存在检测成本高、周期长和效率低等问题。而且检测标准中的大部分化合物在实际样品检测中并未检出过,目标针对性不强。因此,需要整合化妆品中实际添加的多种类禁用物质,建立一种高效、准确、覆盖广、针对性强的化妆品中常见禁用物质的筛查方法,以实现对批量样品的快速筛查。而目前对于化妆品中多种禁用物质的同时检测还鲜有报道。

本研究根据文献^[14⇓⇓⇓-18]^检索结果和监测结果筛选出在市售化妆品中检出过的17大类73种常见禁用物质作为目标分析物,建立了化妆品中73种常见禁用物质的UPLC-Q-TOF HRMS检测方法。此外,在不合格化妆品通告和文献^[14⇓⇓⇓-18]^检索的基础上,还针对性地建立了化妆品中禁用物质高分辨质谱筛查数据库,并将数据库应用于日常化妆品监督检验和风险监测。筛查数据库的应用大大提高了日常检验的工作效率和化妆品安全风险监测水平,对于保障化妆品质量安全、保护消费者健康具有非常重要的现实意义。

## 1 实验部分

### 1.1 仪器、试剂与材料

Exion LC超高效液相色谱仪、X500R四极杆-飞行时间高分辨质谱仪(配有Sciex OS数据处理软件和LibraryView数据库软件)(美国AB Sciex公司), 3-18KS高速冷冻离心机(美国Sigma公司), MS3 basic旋涡振荡器(德国IKA公司), XS205DU电子天平(瑞士Mettler公司), Ti-H20超声波仪(德国Heidolpn公司), Milli-Q超纯水处理系统(美国Millipore公司)。

乙酸铵(质谱纯,美国Sigma公司);甲酸(质谱纯)、甲醇、乙腈(色谱纯)、*N*-丙基乙二胺(PSA)、十八烷基硅烷键合硅胶(C_18_)、无水硫酸镁(优级纯)均购自德国CNW公司。73种禁用物质包括33种激素类(25种糖皮质激素和8种性激素)、18种抗生素类(4种喹诺酮类、4种四环素类、4种磺胺类、3种林可酰胺类、2种硝基咪唑类和1种酰胺醇类)、7种抗真菌类、3种抗组胺类、3种香豆素类、3种局部麻醉类、2种肾上腺素类、1种解热镇痛类、1种育发类、1种抗寄生虫类和1种促进胶原蛋白合成类。73种禁用物质按上述类别顺序和同一类中化合物的保留时间顺序依次列于表1。

**表1:** 73种化合物的品牌、纯度和质谱参数

No.		Compound		Category		*t*_R_/ min		Precursor ion (*m/z*)		Fragment ion (*m/z*)		CE/ eV		Brand	Purity/ %
1		beclometasone (倍氯米松)		glucocorticoids		7.91		409.1838		147.0807		15		TMRM	98.0
2		16*α*-hydroxyprednisolone (16*α*-羟基泼尼松龙)		glucocorticoids		10.82		377.1963		147.0804		30		Bidepharm	97.0
3		prednisone (泼尼松)		glucocorticoids		11.30		359.1851		147.0809		35		NIFDC	99.6
4		cortisone (可的松)		glucocorticoids		11.48		361.1928		163.1110		34		Aladdin	97.8
5		hydrocortisone (氢化可的松)		glucocorticoids		11.86		363.2161		121.0646		23		Aladdin	98.0
6		diflorasone (二氟拉松)		glucocorticoids		12.23		411.1983		121.0656		30		Macklin	99.4
7		meprednisone (甲基泼尼松)		glucocorticoids		12.48		373.2005		355.1892		14		CATO	98.0
8		betamethasone (倍他米松)		glucocorticoids		12.51		393.2074		355.1898		15		Macklin	99.3
9		dexamethasone (地塞米松)		glucocorticoids		12.63		393.2074		355.1898		15		NIFDC	100.0
10		fluocinoloneacetonide (氟轻松)		glucocorticoids		12.72		453.2084		121.0649		24		Aladdin	99.4
11		prednisone 21-acetate (泼尼松醋酸酯)		glucocorticoids		12.79		401.1959		295.1687		23		TRC	98.0
12		triamcinolone acetonide (曲安奈德)		glucocorticoids		12.81		435.2175		415.2107		15		NIFDC	98.8
13		methylprednisolone (甲基泼尼松龙)		glucocorticoids		12.84		375.2169		161.0966		28		Macklin	99.5
14		cortisone acetate (可的松醋酸酯)		glucocorticoids		12.91		403.2106		163.1120		34		Aladdin	98.0
15		desonide (地索奈德)		glucocorticoids		13.32		417.2277		147.0807		39		Aladdin	99.0
16		betamethasone acetate (倍他米松醋酸酯)		glucocorticoids		13.57		435.2181		397.2008		15		USP	99.7
17		dexamethasone-17-acetate (地塞米松醋酸酯)		glucocorticoids		13.80		435.2181		397.2008		15		NIFDC	100.0
18		fluocinonide (氟轻松醋酸酯)		glucocorticoids		14.69		495.2183		337.1432		25		Bepure	98.5
19		triamcinolone acetonide acetate (曲安奈德醋酸酯)		glucocorticoids		14.87		477.2187		339.1593		22		NIFDC	99.1
20		cortisol 17-valerate (氢化可的松戊酸酯)		glucocorticoids		15.63		447.2731		345.2035		19		Bepure	98.3
21		clobetasol propionate (氯倍他索丙酸酯)		glucocorticoids		15.73		467.1999		355.1459		18		Yuanye	98.0
22		mometasonefuroate (莫米他松糠酸酯)		glucocorticoids		15.80		521.1496		503.1370		16		Yuanye	98.0
No.		Compound		Category		*t*_R_/ min		Precursor ion (*m/z*)		Fragment ion (*m/z*)		CE/ eV		Brand	Purity/ %
23		betamethasone 17-valerate (倍他米松戊酸酯)		glucocorticoids		16.14		477.2518		279.1734		18		Aladdin	98.1
24		betamethasone dipropionate (倍他米松双丙酸酯)		glucocorticoids		17.02		505.2536		411.2155		15		Aladdin	97.2
25		beclometasonedipropionate (倍氯米松双丙酸酯)		glucocorticoids		17.72		521.2375		503.2189		16		Bepure	98.8
26		diethylstilbestrol (己烯雌酚)		sex hormones		10.47		267.1436		237.0813		-35		NIFDC	98.9
27		estrone (雌酮)		sex hormones		10.57		269.1480		145.0593		-50		TCI	99.9
28		testosterone (睾丸酮)		sex hormones		14.34		289.2155		97.0639		29		Aladdin	99.2
29		levonorgestrel (炔诺孕酮)		sex hormones		14.85		313.2157		245.1894		24		TRC	98.0
30		17-methyltestosterone (甲睾酮)		sex hormones		15.12		303.2301		97.0640		30		Macklin	98.0
31		megestrol acetate (醋酸甲地孕酮)		sex hormones		16.01		385.2367		325.2135		20		NIFDC	99.2
32		progesterone (黄体酮)		sex hormones		16.55		315.2300		97.0640		32		TCI	99.1
33		testosterone propionate (丙酸睾酮)		sex hormones		19.37		345.2418		97.0643		30		Macklin	98.0
34		ofloxacin (氧氟沙星)		quinolones		7.43		362.1509		318.1590		26		Aladdin	99.6
35		enoxacin (依诺沙星)		quinolones		7.48		321.1355		303.1227		24		Aladdin	98.5
36		ciprofloxacin (环丙沙星)		quinolones		7.82		332.1403		314.1276		25		Aladdin	99.0
37		enrofloxacin (恩诺沙星)		quinolones		7.94		360.1713		342.1593		38		Aladdin	98.2
38		tetracycline (四环素)		tetracycline		7.69		445.1599		410.1240		30		NIFDC	96.9
39		oxytetracycline (土霉素)		tetracycline		7.87		461.1567		426.1182		29		Yuanye	95.0
40		chlortetracycline (金霉素)		tetracycline		9.35		479.1223		462.0945		38		NIFDC	94.1
41		minocycline (美满霉素)		tetracycline		12.86		458.2027		441.1651		22		Dr. Ehrenstorfer	99.5
42		sulfamerazine (磺胺甲基嘧啶)		sulfonamides		6.44		265.0758		156.0114		23		Aladdin	99.0
43		trimethoprim (甲氧苄啶)		sulfonamides		6.95		291.1429		291.1423		25		Macklin	99.9
44		sulfamethizol (磺胺甲噻二唑)		sulfonamides		7.10		271.0320		156.0115		20		Dr. Ehrenstorfer	99.3
45		sulfamethoxazole (磺胺甲基异噁唑)		sulfonamides		7.79		254.0595		156.0114		21		Dr. Ehrenstorfer	99.6
46		lincomycin (林可霉素)		lincosamides		6.88		407.2207		126.1267		27		Yuanye	98.0
47		clindamycin (克林霉素)		lincosamides		10.72		425.1862		126.1261		27		NIFDC	87.2
48		clindamycin phosphate (克林霉素磷酸酯)		lincosamides		15.73		505.1544		126.1270		35		TCI	99.7
49		metronidazole (甲硝唑)		nitroimidazole		5.30		172.0721		128.0456		20		Aladdin	99.8
50		tinidazole (替硝唑)		nitroimidazole		6.39		248.0698		121.0321		25		NIFDC	99.9
51		chloramphenicol (氯霉素)		amphenicol		8.52		321.0038		152.0278		-22		NIFDC	99.3
52		cyproheptadine (赛庚啶)		antifungal		11.73		288.1737		96.0799		31		Aladdin	98.0
53		griseofulvin (灰黄霉素)		antifungal		12.05		353.0777		165.0535		26		Aladdin	97.2
54		ketoconazole (酮康唑)		antifungal		12.29		531.1540		489.1432		43		Aladdin	99.0
55		bifonazole (联苯苄唑)		antifungal		12.50		311.1539		109.0639		23		Bepure	99.7
56		terbinafine (特比萘芬)		antifungal		12.91		292.2039		141.0675		25		Macklin	98.0
57		econazole (益康唑)		antifungal		14.33		381.0306		125.0131		36		Aladdin	99.0
58		miconazole (咪康唑)		antifungal		15.82		414.9918		158.9756		40		NIFDC	98.6
59		cimetidine (西咪替丁)		antihistamines		5.38		253.1224		95.0596		25		NIFDC	99.8
60		chlorpheniramine (氯苯那敏)		antihistamines		10.15		275.1297		230.0699		25		Aladdin	99.0
61		diphenhydramine (苯海拉明)		antihistamines		10.67		256.1698		167.0830		16		Aladdin	99.0
62		6-methylcoumarin (6-甲基香豆素)		coumarin		11.01		161.0594		105.0689		25		CNW	99.0
63		7-methylcoumarin (7-甲基香豆素)		coumarin		11.05		161.0594		105.0689		25		TCI	98.0
64		maraniol (7-乙氧基-4-甲基香豆素)		coumarin		12.49		205.0848		177.0523		25		AlfaAesar	98.0
65		procaine (普鲁卡因)		anaesthesia		5.45		237.1598		100.1108		25		NIFDC	99.2
66		lidocaine (利多卡因)		anaesthesia		7.57		235.1797		86.0951		25		NIFDC	99.4
67		tetracaine (丁卡因)		anaesthesia		10.43		265.1902		176.1046		25		NIFDC	94.0
68		brimonidine (溴莫尼定)		epinephrine		5.45		292.0192		212.0927		25		Yuanye	98.0
69		naphazoline (萘甲唑啉)		epinephrine		8.37		211.1223		141.0694		25		NIFDC	99.2
70		4-acetamidophenol (对乙酰氨基酚)		antipyretic		5.18		152.0709		110.0603		25		NIFDC	99.9
71		minoxidil (米诺地尔)		hair-nutrition		8.12		210.1340		193.1297		21		NIFDC	100.0
72		crotamiton (克罗米通)		antiparasitic		13.45		204.1374		69.0327		25		Macklin	94.7
73		alfacalcidol (阿法骨化醇)		collagen synthesis		25.76		401.3413		95.0860		25		NIFDC	99.8

CE: collision energy.

### 1.2 实验条件

#### 1.2.1 样品前处理

准确称取化妆品样品0.2 g,置于15 mL离心管中,加入3 mL饱和氯化钠溶液,涡旋30 s,分散均匀,加入含0.2%甲酸的乙腈溶液10 mL,涡旋30 s,超声提取30 min,涡旋混合摇匀,于0 ℃以8000 r/min转速冷冻离心5 min,吸取上清液5 mL,置于10 mL离心管中,加入50 mg PSA净化吸附剂涡旋1 min混匀,于0 ℃以8000 r/min转速离心后,取上清液经0.22 μm滤膜过滤后,上机测定。

#### 1.2.2 标准溶液的配制

分别精确称取10 mg对照品,置于10 mL棕色容量瓶中,加入甲醇充分溶解后(对于在纯甲醇中溶解性差的环丙沙星、美满霉素、磺胺甲基嘧啶、磺胺甲噻二唑和金霉素可加入少量甲酸或水促进溶解),室温超声10 min,用甲醇定容至刻度,摇匀,配制成质量浓度为1000 mg/L的标准储备液,置于-18 ℃冰箱中储存。用甲醇将标准储备液逐级稀释成不同质量浓度的标准工作液。

取15 mL离心管8支,每支称取与待测化妆品配方相同或相近的基质空白样品0.2 g,分别精密加入标准工作液适量,涡旋30 s混匀,按1.2.1节样品前处理步骤进行处理,配制成含量为50~10000 μg/kg的系列基质匹配标准溶液。

#### 1.2.3 色谱条件

色谱柱:Waters Acquity HSS T3色谱柱(100 mm×2.1 mm, 1.8 μm);柱温:40 ℃;流速:0.35 mL/min。正离子模式流动相:含0.1%甲酸的5 mmol/L乙酸铵溶液(A)和含0.1%甲酸的甲醇(B);正离子模式梯度洗脱程序:0~1.0 min, 2%B; 1.0~3.0 min, 2%B~20%B; 3.0~8.0 min, 20%B~40%B; 8.0~15.0 min, 40%B~60%B; 15.0~20.0 min, 60%B~80%B; 20.0~25.0 min, 80%B~95%B; 25.0~27.0 min, 95%B; 27.0~27.5 min, 95%B~2%B; 27.5~30.0 min, 2%B。负离子模式流动相:0.005%甲酸水溶液(C)和含0.005%甲酸的甲醇(D);负离子模式梯度洗脱程序:0~0.5 min, 2%D; 0.5~14.0 min, 2%D~98%D; 14.0~17.5 min, 98%D; 17.5~17.6 min, 98%D~2%D; 17.6~20.0 min, 2%D。进样量:5 μL。

#### 1.2.4 质谱条件

离子源:电喷雾电离(ESI)源;离子化方式:ESI^+^和ESI^-^;毛细管温度:600 ℃;雾化气(gas 1)压力:379.21 kPa(55 psi);干燥气(gas 2)压力:379.21 kPa (55 psi);气帘气(curtain gas)压力:241.32 kPa (35 psi);碰撞气(CAD gas)压力:48.26 kPa (7 psi);监测模式:多反应监测高分辨扫描模式(MRM HR);喷雾电压:5.5/-4.5 kV;去簇电压(DP):±80 V;一级全扫描模式:全扫描范围*m/z* 100~1000;二级MRM HR模式:扫描范围*m/z* 60~600。73种化合物的母离子、子离子、碰撞能量(CE)、保留时间、标准物质纯度等参数列于表1。

每次实验前,利用厂家提供的正、负离子调谐液,通过内置调谐液传输系统(CDS)对仪器分别进行MS和MS/MS模式的质量精度校正。序列进行中,每运行5针样品,利用厂家提供的校正液自动进行一次质量精度校正。

## 2 结果与讨论

### 2.1 高分辨质谱筛查数据库的建立和应用

采用AB X500R Q-TOF构建化妆品中禁用物质高通量风险筛查质谱数据库,目前已通过购买标准品自建了化妆品中650种禁用物质的高通量风险筛查识别数据库,并应用于日常化妆品的风险监测。采用信息依赖扫描模式(IDA)建立数据库并应用于样品的快速筛查。通过数据库中化合物的保留时间、母离子及碎片离子精确质量信息、同位素丰度比等对样品中的禁用物质进行快速筛查和定性确证。如果被测样品出现了与标准品保留时间一致的色谱峰、精确质量数偏差小于5×10^-6^、同位素丰度比差异小于5%、二级质谱图谱库匹配得分大于70分时,则可判断被测样品中存在相应的化合物^[19]^。

以克罗米通的快速筛查确证为例,对未知样品进行IDA模式下的一级、二级全扫描检测,将检测结果通过Library View软件与自建数据库进行比对筛查和确证,结果筛查出克罗米通,匹配度为99.5%。克罗米通的提取离子色谱图、一级、二级高分辨质谱图及数据库匹配结果见图1。

**图1:**
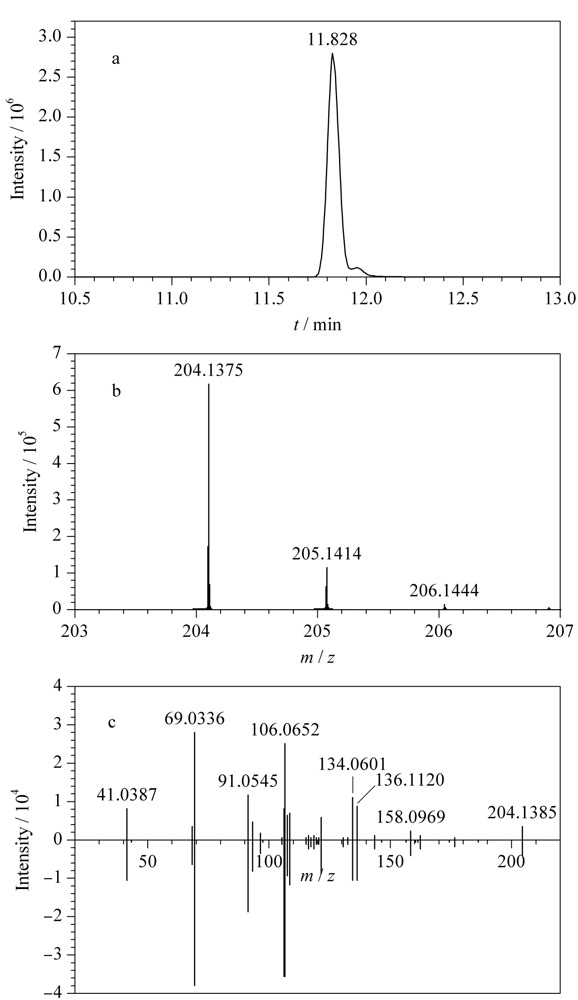
克罗米通的(a)色谱图、(b)一级、(c)二级高分辨质谱图 及数据库镜像结果

### 2.2 质谱条件选择

根据73种化合物的电离性质,分别选用ESI^+^和ESI^-^作为离子化模式,采用流动注射泵连续进样方式进行一级质谱全扫描,选择响应最强的目标离子作为一级母离子,同时对去簇电压、碰撞能量等质谱参数进行优化。氯霉素、雌酮和己烯雌酚均在负离子模式下响应最大,准分子离子为[M-H]^-^;其余化合物均在正离子模式下响应最大,准分子离子为[M+H]^+^。本文使用的飞行时间质谱可以根据需要选择IDA模式、连续窗口全理论碎片离子采集模式(SWATH)和高分辨多反应监测模式(MRM HR)等3种不同的扫描模式。其中,对于已知化合物的定量,选择MRM HR模式响应最好,并可通过根据化合物保留时间分段采集和设置最佳CE值来获得质谱最佳响应。相较于IDA模式,MRM HR模式下,73种禁用物质的质谱响应可提高约1.3~37倍。

### 2.3 色谱条件选择

实验考察了不同流动相组成(水-甲醇、0.1%甲酸水溶液-甲醇、含0.1%甲酸的5 mmol/L乙酸铵溶液-含0.1%甲酸的甲醇)对待测化合物分离和质谱响应的影响。流动相中酸度越强,正离子模式下化合物响应越高,负离子模式下化合物响应受抑制。综合考虑待测组分的化学性质、色谱分离和质谱响应,最终选择正离子模式下含0.1%甲酸的5 mmol/L乙酸铵溶液和含0.1%甲酸的甲醇溶液作为流动相。负离子模式下0.005%甲酸水溶液和含0.005%甲酸的甲醇溶液作为流动相。

实验对梯度洗脱程序进行了优化,使得不同类别、不同性质的化合物能够较好地色谱分离,且保留时间分布较均匀。

由于17类73种常见禁用物质的分子结构和化学性质差异较大,本研究考察了73种禁用物质在Waters Acquity HSS T3(100 mm×2.1 mm, 1.8 μm)、Agilent Poroshell 120 SB-AQ(100 mm×2.1 mm, 2.7 μm)和Agilent Poroshell 120 EC-C18(100 mm×2.1 mm, 2.7 μm)3款通用型色谱柱上的色谱行为(见附图1,详见 http://www.chrom-China.com)。实验结果表明,73种禁用物质在3款色谱柱上的色谱峰均对称性好,峰形窄。相较于SB-AQ和EC-C18色谱柱,大多数化合物在HSS T3色谱柱上保留时间延长,且质谱响应信号也明显增强,有利于目标分析物与样品中杂质的有效分离。Acquity HSS T3色谱柱采用亚微米填料技术,比传统的色谱柱具有更好的色谱分离度,特别可增强极性化合物的色谱保留。因此,本实验最终选择HSS T3色谱柱用于73种禁用物质的色谱分离。73种禁用物质的提取离子色谱图见图2。

**图2:**
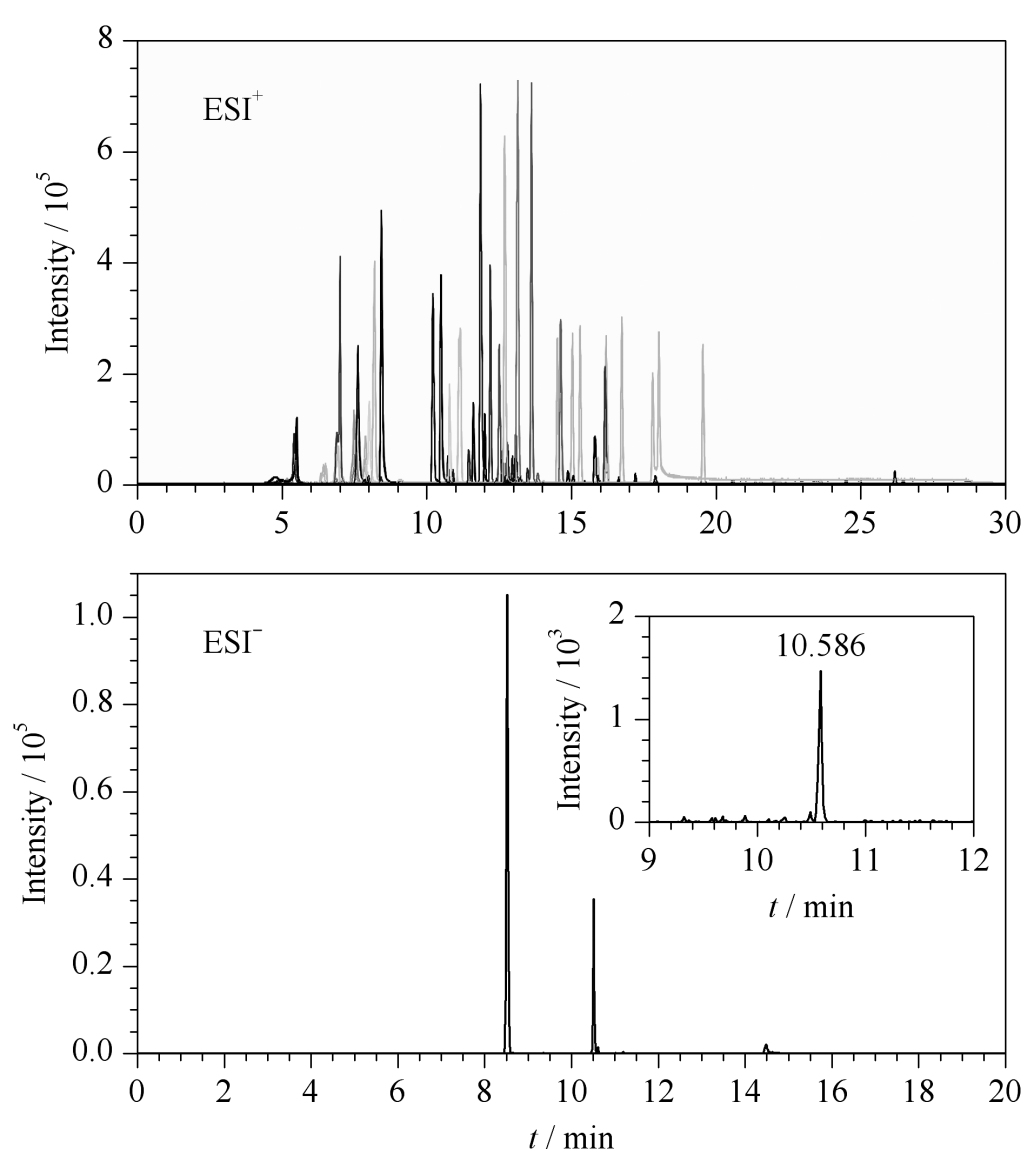
73种禁用物质的提取离子色谱图

### 2.4 样品前处理条件的优化

#### 2.4.1 提取溶剂的选择

73种禁用物质中大部分化合物在甲醇、乙腈中有较好的溶解性,但环丙沙星、美满霉素、磺胺甲基嘧啶和磺胺甲噻二唑需一定比例的甲醇水溶液或乙腈水溶液才能完全溶解,金霉素还需加入少量甲酸助溶。本研究所涉及的73种禁用物质包含多种不同性质的化合物,化学性质差异较大,需要对提取溶剂进行选择和优化。对比甲醇和乙腈的提取效果,发现当用甲醇作为提取溶剂时,样品起泡现象较乙腈明显,且甲醇与起破乳作用的饱和氯化钠溶液互溶,不易除盐。而乙腈不与饱和氯化钠溶液互溶,且乙腈有沉淀蛋白质的作用,有利于除去样品中蛋白质等杂质。此外,据文献^[19,20]^报道,磺胺类物质大多适宜用碱性提取,而四环素类、喹诺酮类化合物在酸碱两性条件下提取效果较好,回收率高。因此实验考察了纯乙腈和含不同体积分数甲酸(0.1%、0.2%、0.5%和1.0%)、氨水(0.1%和0.2%)的乙腈溶液提取溶剂对膏霜类样品中待测组分提取回收率的影响(见图3),通过代入溶剂标准曲线计算提取回收率。

**图3:**
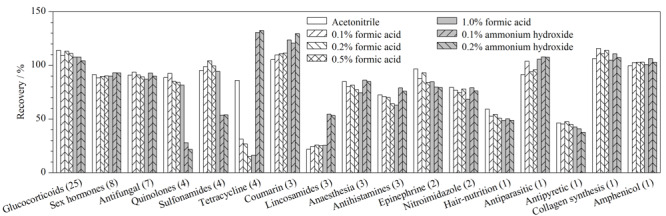
提取溶剂对73种禁用物质回收率的影响

因73种化合物数量众多,本研究将同一类化合物的回收率求均值后按类进行比较。结果表明,大多数化合物的提取不受酸碱性条件的影响,但加入一定量的甲酸可提高待测物的离子化效率,随着甲酸体积分数的增加,喹诺酮类和磺胺类物质提取回收率先增加后降低,酸性条件下的提取回收率显著高于碱性条件下;但对于林可酰胺类和美满霉素,则碱性条件下的提取回收率显著高于酸性条件,美满霉素随着酸度的增加,提取回收率逐渐降低。综合多组分的提取效果,最终选用对于绝大多数化合物提取回收率较高的0.2%甲酸乙腈溶液作为提取溶剂。

本实验采用饱和氯化钠溶液对化妆品样品进行加速破乳,但林可酰胺类等水溶性化合物因分布在水层而导致回收率低于30%,林可霉素回收率更是低于10%(见图3),林可霉素较低的回收率与文献^[19]^结果一致。因此,针对林可酰胺类物质,如采用饱和氯化钠溶液破乳,则需采用基质匹配外标法进行定量。如果待测化妆品属于“油包水”剂型,还可加入适量的四氢呋喃或异丙醇进行破乳,以提高目标化合物的回收率。

由于化妆品基质较为复杂,考察了涡旋分散和超声的提取方法,涡旋分散有利于样品与提取溶剂充分接触,超声对于化合物的提取效率更高,两者结合操作简便,提取效率更好。采用高速冷冻离心的方式净化除杂,可快速简便地将提取溶液与化妆品基质中的脂类等大分子物质有效分离。

#### 2.4.2 净化吸附剂的选择

QuEChERS的原理是利用固体吸附剂选择性吸附杂质从而达到净化样品的目的,因此吸附剂的选择和用量是一个重要的考察因素。常用净化剂一般包括PSA、C_18_和无水硫酸镁。PSA是同时含有伯胺和仲胺基团的高纯硅胶基质类极性吸附剂,具有极性作用和弱阴离子交换作用,可有效去除有机酸、脂肪和色素等水溶性杂质。C_18_是一种憎水硅胶基吸附剂,能够吸附脂肪等非极性干扰物。无水硫酸镁在净化过程中可吸取多余的水分。

本研究比较了PSA、C_18_和硫酸镁3种净化吸附剂对化妆品中73种常见禁用物质的净化效果。选择基质更为复杂的膏霜样品为考察基质,样品加入200 ng混合标准溶液后按前处理方法提取,以17大类禁用物质的平均回收率为考察指标,研究了PSA(0、50、100、200和500 mg)、C_18_(0、50、100和200 mg)和硫酸镁(0、100、200、500、1000和1500 mg)用量对于各类化合物回收的影响(见图4)。

**图4:**
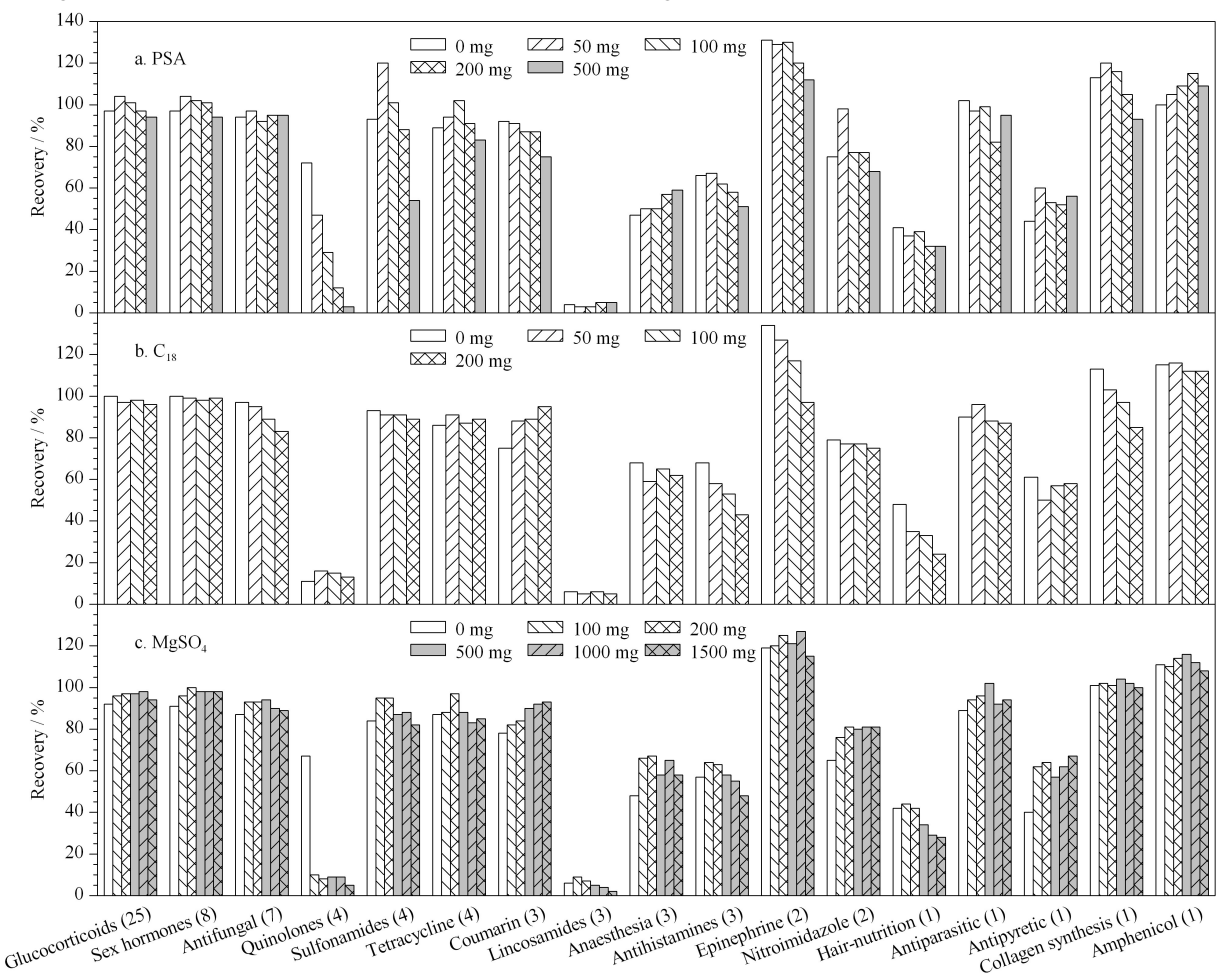
净化吸附剂对73种禁用物质回收率的影响

每个条件均进行2次平行实验,2次平行测定的相对偏差均小于15%。由图4a可知,PSA对于喹诺酮类化合物具有明显吸附作用,回收率随着PSA用量的增加而显著降低,与陈少波等^[21]^研究结果一致。糖皮质激素、磺胺类、抗组胺类、肾上腺素类、阿法骨化醇等化合物均随着PSA用量增加回收率降低,当PSA用量为50 mg时,对绝大多数化合物在有净化效果的同时保证了较好的回收。空白膏霜基质经50 mg的PSA净化前后的总离子流图见图5。由图可见,50 mg的PSA具有很好的净化效果,可大大降低基质干扰。

**图5:**
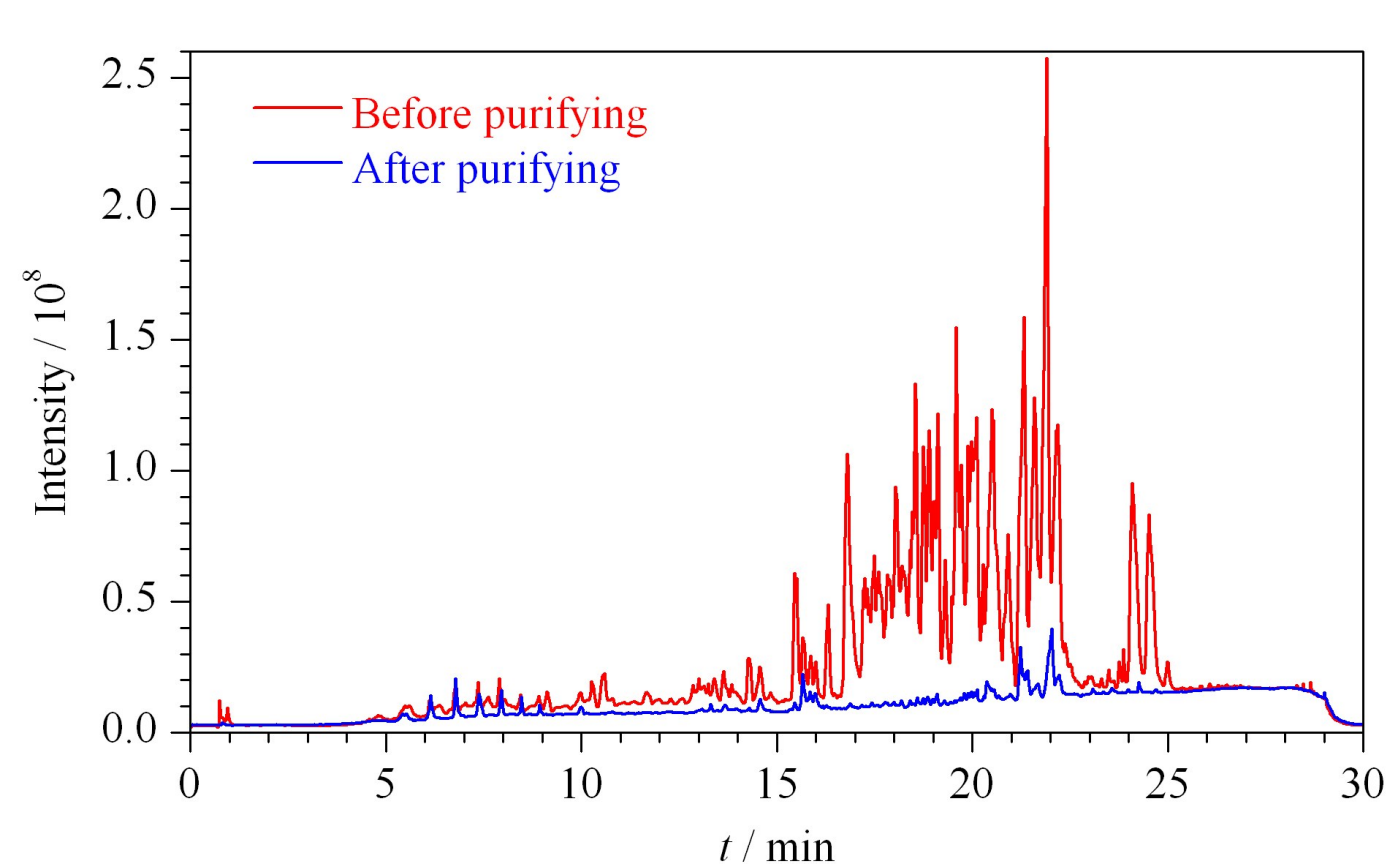
采用PSA净化前后膏霜剂样品的总离子流色谱图

由图4b可知,C_18_对抗真菌类、抗组胺类、肾上腺激素类、米诺地尔、阿法骨化醇有一定的吸附作用,回收率随着C_18_用量的增加而降低,且对大多数化合物,C_18_用量对化合物的回收影响不大,因此不采用C_18_作为净化剂。

无水硫酸镁可作为盐析剂和除水剂,有利于有机相和水相分层,防止水分和杂质进入提取液,在商品化的QuEChERS净化管中常与C_18_和PSA联合使用。文献^[22]^报道硫酸镁可能与喹诺酮类化合物吸附结合,形成配合物,导致回收率低于10%,本研究中喹诺酮类化合物回收率较低与文献结果一致,但硫酸镁对其他类化合物的影响不显著(见图4c)。因此,不采用无水硫酸镁作为净化剂。

综合各化合物的回收效果,本研究最终选择50 mg PSA。

### 2.5 基质效应考察

基质效应(*η*)是指共流出干扰物对目标物离子造成离子抑制或增强的效应,基质效应在质谱分析中普遍存在,影响分析结果的准确性。本研究选取膏霜剂和水剂作为典型基质对基质效应进行考察,分别制备溶剂标准溶液、膏霜剂和水剂的基质匹配标准溶液,测得溶剂标准曲线和2种基质匹配标准曲线性方程的斜率。基质效应=(基质匹配标准曲线斜率-溶剂标准曲线斜率)/溶剂标准曲线斜率×100%^[23]^, *η*的绝对值随着基质效应的增强而变大,*η*为正值说明基质增强,负值则为基质抑制。当*η*绝对值<20%时表示基质效应微弱,*η*绝对值在20%~50%范围时表示具有中等强度基质效应,*η*绝对值>50%时表示基质效应较强^[24]^。膏霜剂和水剂中73种禁用物质的*η*值见附表1,由附表1可知,水剂*η*值为-95.4%~-0.2%,膏霜剂*η*值为-96.3%~-8.9%。水剂基质中50%的化合物都具有中等以上强度的基质抑制效应,膏霜基质中88%的化合物都具有中等以上强度的基质抑制效应。因此,本实验采用基质匹配工作溶液定量以减小基质效应的影响。

### 2.6 线性范围、检出限和定量限

本实验分别以水剂和膏霜剂化妆品空白样品为基质,对8个含量在50~10000 μg/kg(部分化合物对5个含量在500~10000μg/kg)之间的系列混合基质标准工作溶液进行测定,以化合物含量为横坐标(μg/kg),以峰面积为纵坐标绘制基质匹配标准曲线。结果表明,各化合物两种基质下在相应范围内线性关系良好,相关系数(*R*^2^)均大于0.99。以信噪比(*S/N*)≥3确定检出限,为5~150 μg/kg;以*S/N*≥10确定定量限,为15~450 μg/kg。73种禁用物质水剂和膏霜剂的线性范围、检出限和定量限见附表1。

### 2.7 回收率和精密度

分别选取膏霜和水剂空白基质样品,做3个添加水平(LOQ、5倍LOQ、10倍LOQ)的加标回收试验,每个添加水平分别连续测定6次和连续3 d每天连续测定6次,计算各待测物的平均回收率及日内和日间相对标准偏差(见附表2)。结果表明,73种禁用物质在膏霜剂及水剂两种基质中3个加标水平下的平均加标回收率为60.3%~130.3%,日内RSD为0.8%~10.0%(*n*=6),日间RSD为1.1%~15.0%(*n*=3)。

### 2.8 实际样品测定

应用本实验所建立的方法对日常化妆品风险监测所抽取的692份化妆品样品进行分析。从其中16个样品中检出16种禁用物质,检出的禁用物质有磺胺甲基异噁唑、甲基泼尼松、林可霉素、对乙酰氨基酚、甲氧苄啶、阿法骨化醇、倍他米松戊酸酯、溴莫尼定、氯霉素、氯苯那敏、氯倍他索丙酸酯、克罗米通、益康唑、酮康唑、泼尼松醋酸酯和泼尼松,检出禁用物质的含量范围为0.5~1136.1 mg/kg,每个样品平行测定2份,2份平行样品的相对偏差均小于10%,样品的测定结果见附表3。当样品中被测组分的含量超过基质匹配标准曲线范围后,用含0.2%甲酸的乙腈溶液对样品提取液进行适当稀释后进行检测。其中,阿法骨化醇、对乙酰氨基酚、甲基泼尼松、甲氧苄啶、克罗米通和溴莫尼定等6种化合物为现行检测标准外的禁用物质。

## 3 结论

本文采用UPLC-Q-TOF HRMS建立了化妆品中73种常见禁用物质的快速筛查确证方法。经实际样品检测考察,该方法准确、快速、简便、实用,适用于化妆品中常见禁用物质的同时检测,具有较强的实际应用价值,大大提高了日常监督检验和风险监测的检验效率,降低了检测成本,方法的各项技术指标也满足现行标准要求。同时,本研究还建立了化妆品中安全风险物质高分辨质谱筛查数据库,并将其应用于日常的监管工作。数据库的建立和应用将为化妆品监管提供新工具和新方法,有助于实现化妆品安全风险的及时监测、准确研判、科学预警和有效处置。

## References

[1] Luo H T, Huang X L, Wu H Q, et al. Chinese Journal of Analytical Chemistry, 2017, 45(9): 1381

[2] Chen J, Zheng R, Mao B P, et al. Flavour Fragrance Cosmetics, 2020(6): 80

[3] Sun S S, Yang Y, Ma M, et al. Flavour Fragrance Cosmetics, 2019(5): 26

[4] Yang P P, Liu H, Li L X. China Surfactant Detergent﹠Cosmetics, 2021, 51(5): 468

[5] Li H Y, Shen H D, Fang J J, et al. Chinese Journal of Chromatography, 2018, 36(7): 643 30136536 10.3724/SP.J.1123.2018.01041

[6] National Food and Drug Administration. Safety and Technical Standards for Cosmetics (2015 Edition). Beijing: People's Medical Publishing House, 2015

[7] GB/T 24800.2-2009

[8] GB/T 32986-2016

[9] Niu Z Y, Luo X, Wang F M, et al. Journal of Chinese Mass Spectrometry Society, 2016, 37(3): 201

[10] Wang M Y, Chen Y C, Tu F Q, et al. Chinese Journal of Chromatography, 2020, 38(12): 1423 34213257 10.3724/SP.J.1123.2020.06017

[11] Lan C, Shao L Z, Xu J, et al. Physical Testing and Chemical Analysis Part B: Chemical Analysis, 2020, 56(6): 686

[12] Lü W, Li H Y, Li L X, et al. Physical Testing and Chemical Analysis Part B: Chemical Analysis, 2021, 57(5): 470

[13] Li J, Zhou Z M. Flavour Fragrance Cosmetics, 2021(1): 26

[14] Wang C, Lu J, Guo C M. Detergent & Cosmetics, 2020, 43(4): 36

[15] Wang C, Dong G, Li L, et al. Journal of Instrumental Analysis, 2020, 39(6): 756

[16] Lei Y, He J W, Huang Y T, et al. Journal of Instrumental Analysis, 2013, 32(3): 326

[17] Wang S, Li Q, Wu X J. Flavour Fragrance Cosmetics, 2020(2): 48

[18] Liang W Y, Li L, Xia Z M, et al. Flavour Fragrance Cosmetics, 2021(1): 22

[19] Guo H X, Xiao G Y, Zhang X Q, et al. Chinese Journal of Chromatography, 2015, 33(12): 1242 27097457 10.3724/sp.j.1123.2015.09016

[20] Chen X, Wu C, Wang L X, et al. Chinese Journal of Chromatography, 2018, 36(11): 1147 30378378 10.3724/SP.J.1123.2018.06029

[21] Chen S B, Pu Y Y, Wang H R, et al. Journal of Instrumental Analysis, 2018, 37(11): 1302

[22] Gao F D, Zhao Y, Shao B, et al. Chinese Journal of Chromatography, 2012, 30(6): 560 23016288 10.3724/sp.j.1123.2012.02021

[23] Dong M F, Bai B, Tang H X, et al. Chinese Journal of Analytical Chemistry, 2015, 43(5): 663

[24] Meng M H, He Z Y, Xu Y P, et al. Journal of Agro-Environment Science, 2017, 36(8): 1672

